# Distribution of Sentinel Nodes in Non‐Parotid Salivary Gland Tumors—A Feasibility Study

**DOI:** 10.1002/cnr2.70510

**Published:** 2026-03-06

**Authors:** Marcus Jansson, Rusana Bark, Alexandra Elliot, Lalle Hammarstedt‐Nordenvall, Caroline Gahm

**Affiliations:** ^1^ Department of Otorhinolaryngology Karolinska University Hospital Stockholm Sweden; ^2^ Department of Clinical Sciences Intervention and Technology, Division of Ear Nose and Throat Diseases Karolinska Institute Stockholm Sweden; ^3^ Medical Unit Head Neck Lung and Skin Cancer, Department of Head and Neck Surgery Karolinska University Hospital Stockholm Sweden

**Keywords:** micrometastasis, neck treatment, salivary gland tumors, sentinel node

## Abstract

**Background:**

Salivary gland carcinomas are rare and heterogeneous malignancies, more common in the parotid gland but also in submandibular and minor salivary glands. Surgery, often combined with radiotherapy, is the main treatment. Management of the clinically node‐negative (cN0) neck is debated, with some favoring extensive surgery and others active surveillance. The sentinel node (SN) technique may improve nodal staging and personalized surgery but is understudied in non‐parotid tumors.

**Aims:**

This study aimed to evaluate whether the technique is applicable to assess SN distribution in these patients.

**Methods and Results:**

In this prospective observational feasibility study, 40 patients with cytologically suspected benign or malignant non‐parotid salivary gland tumors were enrolled. Preoperatively, patients received an ultrasound‐guided intratumoral injection of Tc‐99m‐labeled tracer, followed by SPECT–CT imaging for SN localization. Intraoperatively, SNs were detected using a gamma probe, and sentinel lymph node biopsy (SLNB) was performed when indicated. SNs were detected in 33 of 39 (85%) patients. Bilateral distribution of SNs was seen in two of 12 patients with minor salivary gland tumors but in none with submandibular tumors (*n* = 27). One patient was excluded due to a non‐salivary gland diagnosis. SLNB was performed in 16 patients with cytology indicating aggressive malignancies in which micrometastases were detected in three patients with submandibular gland tumors and in two patients with minor salivary gland tumors.

**Conclusion:**

SN mapping was feasible in patients with non‐parotid salivary gland tumors, with a high detection rate using SPECT–CT. SN distribution was primarily ipsilateral but bilateral in two patients with minor salivary gland tumors. Identification of SN micrometastases highlights the potential of SLNB to improve staging accuracy. Our findings support further investigation of the SN technique as a diagnostic tool in the management of cN0 neck in this rare tumor group.

## 1 Introduction

Salivary gland carcinomas are rare disease entities, characterized by considerable histopathological variability [1]. They account for around 3%–6% of all malignancies in the head and neck region [2, 3], with tumors in the parotid gland representing the largest subgroup. Less commonly, these tumors originate from the submandibular glands (5%–15%), the sublingual glands, or from minor salivary glands distributed throughout the oral cavity [4, 5, 6].

The treatment of these tumors is mainly based on surgery. Radiotherapy is mainly delivered in a postoperative setting, or when the primary tumor is deemed unresectable. While the treatment of the local disease is subject to some consensus, the extent of regional treatment, including watch‐and‐wait, is a subject under debate. When the patient is diagnosed with cN+ status, the need for a neck dissection including levels I–V is not controversial. However, in the clinically node‐negative tumor, some authors advocate for a more aggressive approach with a selective neck dissection upfront based on the uncertainty of preoperative assessment [7, 8], whereas others advocate for a watch‐and‐wait strategy based on T status and histopathological grading [9]. In addition, there are data from studies indicating a high prevalence of occult regional metastatic disease in patients with tumors of non‐parotid origin [10, 11, 12].

Recently, the use of sentinel node (SN) technique in head and neck cancer has gained more interest. This technique is a well‐established method for detecting early lymph node metastases in, for example, breast cancer, malignant melanoma and oral cancer [13, 14, 15]. However, the use of the SN technique as a precision diagnostic tool in salivary gland cancer is poorly studied. There have been data published on the feasibility of the SN technique in parotid and minor salivary gland tumors but reports on submandibular gland tumors are still lacking [16, 17, 18]. Additionally, bilateral neck metastasis sometimes occurs in salivary gland tumors in the oral cavity or base of tongue, but the frequency of contralateral SNs is unknown. Hence, there is a need for further development in this field. The aim of this study was to evaluate the feasibility of the SN technique and to characterize the individual lymphatic drainage pattern in non‐parotid salivary gland tumors. This approach seeks to enhance our understanding of their metastatic pathways. The technique has the potential to improve staging accuracy, thereby facilitating individualized surgical treatment and reducing the risk of under‐ and overtreatment.

## 2 Materials and Methods

### 2.1 Study Design and Eligibility Criteria

About 40 consecutive patients with cytologically diagnosed non‐parotid salivary gland tumors who met the inclusion criteria and underwent surgical treatment at Karolinska University Hospital, Sweden, between 2020 and 2025 were enrolled in the study. Patients were eligible for inclusion if they met the following criteria: (1) cytologically verified stage cN0 tumor or benign tumor located in any salivary gland other than the parotid gland, provided the tumor was considered resectable; (2) Swedish‐ or English‐speaking with willingness to participate in the study; (3) age over 18 years; and (4) no present contraindications for surgical treatment under general anesthesia. Patients with a history of prior surgery or radiotherapy to the relevant salivary gland were excluded from the study.

In all patients, tumor and nodal status were assessed by clinical examination and fine‐needle aspiration cytology (FNAC). Computed tomography (CT) was performed in cases (*n* = 18) with cytology suggestive of malignancy to enable accurate nodal staging, and magnetic resonance imaging (MRI) was additionally obtained in selected cases only to facilitate surgical planning. Sentinel lymph node biopsy (SLNB) was performed in 16 patients with cytologically suspected high‐grade tumor or highly unclear cytology, with atypical cellular features suggestive of an increased risk of malignancy, a high Ki‐67 proliferative index, and/or cytological features consistent with specific malignant salivary gland tumor subtypes. Cases with indeterminate FNAC were managed according to a multidisciplinary assessment integrating clinical findings, imaging characteristics, and cytological features. When indeterminate FNAC findings were associated with clinical or radiological features suggestive of aggressive behavior, patients were managed according to the extended protocol and included in the SLNB analysis.

### 2.2 SN Detection and Surgery

Prior to tumor removal, patients underwent a preoperative ultrasound‐guided intratumoral injection of a Technetium‐99m (Tc‐99m)‐labeled tracer. Tilmanocept (Lymphoseek, Cardinal Health) was administered only in the first enrolled patient. From February 2021 onward, Nanocolloid (Nanocoll, GE Healthcare) replaced Tilmanocept and was used for all remaining patients. Imaging with a standardized SPECT–CT was performed no earlier than 1 h after intratumoral injection, covering the head, neck, and thorax. Surgery took place within 24 h after tracer administration. Localization of Tc‐99m was intraoperatively confirmed using a gamma probe (EuroProbe, Euromedical Instruments). During surgery, gamma probe measurements were performed on both ipsilateral and contralateral neck levels I–V. Neck levels were defined using the American Head & Neck Society/AAO‐HNS standard classification. The perioperative protocol included the following data: amount of injected radionuclide, time interval between radionuclide injection and SPECT–CT imaging, type of surgery performed (tumor excision solely or combined with SN‐guided neck dissection, specifying the involved neck levels), perioperative localization of the tumor (submandibular gland, buccal region, retromolar trigone, hard palate, soft palate, tonsil or floor of the mouth), and neck levels (I–V) where SNs were detected using gamma probe.

In cases where SLNB was indicated, an extended protocol was applied. Patients received an additional ultrasound‐guided intratumoral injection of Indocyanine Green (ICG), a fluorescent tracer (Verdye, Diagnostic Green GmbH), approximately 30 min prior to surgery. Intraoperative visualization was performed using optical fluorescence imaging with integrated lighting and an HD camera system (Stryker SPY‐PHI). Identified SNs were surgically removed, marked with sutures and preserved in formalin for subsequent histopathological analysis in accordance with Karolinska University Hospital SN protocol. Each SN was sliced into 3 mm thick sections, after which each section was serially cut, with analysis performed on one section every 250 μm.

### 2.3 Statistical Analysis

Descriptive data were presented in counts and percentages. SN distribution was analyzed in relation to tumor size and tumor type using two‐sided Fisher's exact test. A *p* value < 0.05 was considered statistically significant.

## 3 Results

### 3.1 Diagnostic Accuracy of Preoperative FNAC

**FIGURE 1:**
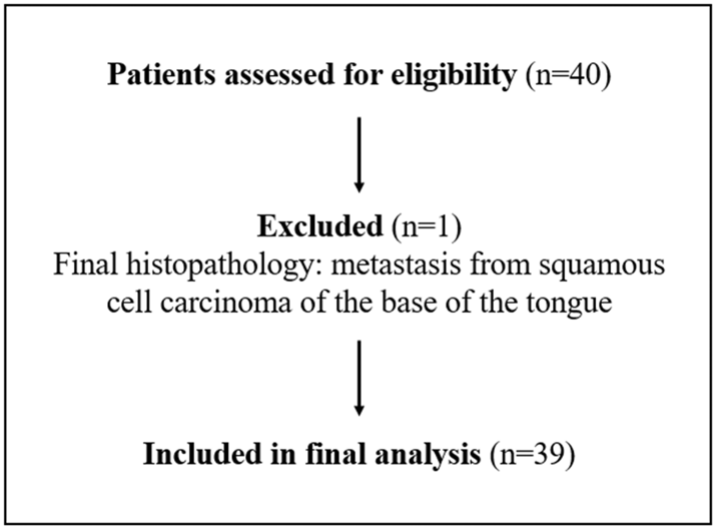
Flow diagram of patient inclusion and exclusion.

A total of 40 consecutive patients were initially enrolled in the study. One patient was subsequently excluded after histopathological analysis revealed a metastasis from a squamous cell carcinoma originating from the base of the tongue, hence not a salivary gland tumor. Therefore, 39 patients were included in the final analysis. A flow diagram of patient inclusion is shown in Figure 1. Patient demographics, tumor size, and tumor location are presented in Table 1. Preoperative FNAC results suggested a benign salivary gland tumor in 16 of 39 patients, a malignant salivary gland tumor in 18 of 39 patients and an indeterminate diagnosis in 5 of 39 patients.

**TABLE 1:** Patient characteristics, tumor size, tumor location, and histopathological diagnosis.

	*N*/value (%)
Gender	
Male	18 (46)
Female	21 (54)
Age (years)	
Min	20
Max	85
Median	54
Tumor size (pT)	
< 2 cm	22 (56)
**≥** 2 cm	17 (44)
Tumor location	
Submandibular gland	27 (69)
Minor salivary gland	12 (31)
Hard palate	5
Soft palate	3
Bucca	1
Tonsils	1
Floor of the mouth	1
Retromolar trigone	1
Histopathological diagnosis	
Pleomorphic adenoma	14
Adenoid cystic carcinoma	7
Adenocarcinoma	4
Mucoepidermoid carcinoma	3
Carcinoma ex pleomorphic adenoma	3
Oncocytoma	2
Salivary duct carcinoma	1
Acinic cell carcinoma	1
Carcinoma in situ	1
Myoepithelioma	1
Inflammation	1
Mucocele	1

*Note:* Percentages are shown for main categories; subcategories are presented as counts only.

The preoperative FNAC diagnosis was consistent with the histopathologic diagnosis in 33 of 39 evaluable patients, and discordant in 6 of 39 patients. Among the discordant cases, three patients were confirmed to have malignant salivary gland tumors upon histopathological examination; however, the tumor subtype differed from that suggested by FNAC. In the remaining three cases, the FNAC diagnoses were interpreted as benign, while histopathological analysis revealed malignant lesions—more specifically, one lesion initially classified as an unclear/pleomorphic adenoma was diagnosed as adenocarcinoma, another lesion suspected to be an unclear but benign tumor was identified as carcinoma ex pleomorphic adenoma, and a third one classified as pleomorphic adenoma was diagnosed as adenoid cystic carcinoma.

### 3.2 Tumor Staging

Among the 18 patients with malignancies as determined by FNAC, TNM staging was as follows: eight patients were staged as T1N0M0, including three with adenoid cystic carcinoma, three with mucoepidermoid carcinoma, one with carcinoma otherwise not specified, and one with carcinoma ex pleomorphic adenoma. Six patients were staged as T2N0M0, comprising two with adenocarcinoma, two with adenoid cystic carcinoma, one with salivary duct carcinoma and one with mucoepidermoid carcinoma. Three patients were staged as T3N0M0 (adenocarcinoma, adenoid cystic carcinoma, and mucoepidermoid carcinoma, respectively), and one patient was staged as T4N0M0 (adenoid cystic carcinoma).

### 3.3 SN Detection and Distribution

The distribution of SNs is shown in Table 2. SNs were identified in neck levels I–V.

**TABLE 2:** Distribution of SNs in relation to tumor site and size.

	All sites *n* (%)	Tumor in submandibular gland	Tumor in minor salivary gland	Tumor < 2 cm	Tumor ≥ 2 cm
No. of patients	39	27	12	22	17
SN level I	4 (10)	3 (11)	1 (8)	2 (9)	2 (12)
SN level II	22 (56)	13 (48)	9 (75)	13 (59)	9 (53)
SN level III	13 (33)	10 (37)	3 (25)	10 (45)	3 (18)
SN level IV	8 (21)	8 (30)	0 (0)	5 (23)	3 (18)
SN level V	1 (3)	0 (0)	1 (8)	0 (0)	1 (6)
SN in one neck level only	19 (49)	14 (52)	5 (42)	8 (36)	11 (65)
SN not detected	6 (15)	4 (15)	2 (17)	3 (14)	3 (18)
SN contralateral side^a^	2 (5)	0 (0)	2 (17)	1 (3)	1 (3)

Abbreviation: SN, sentinel node.

^a^ SN level II and III.

**FIGURE 2:**
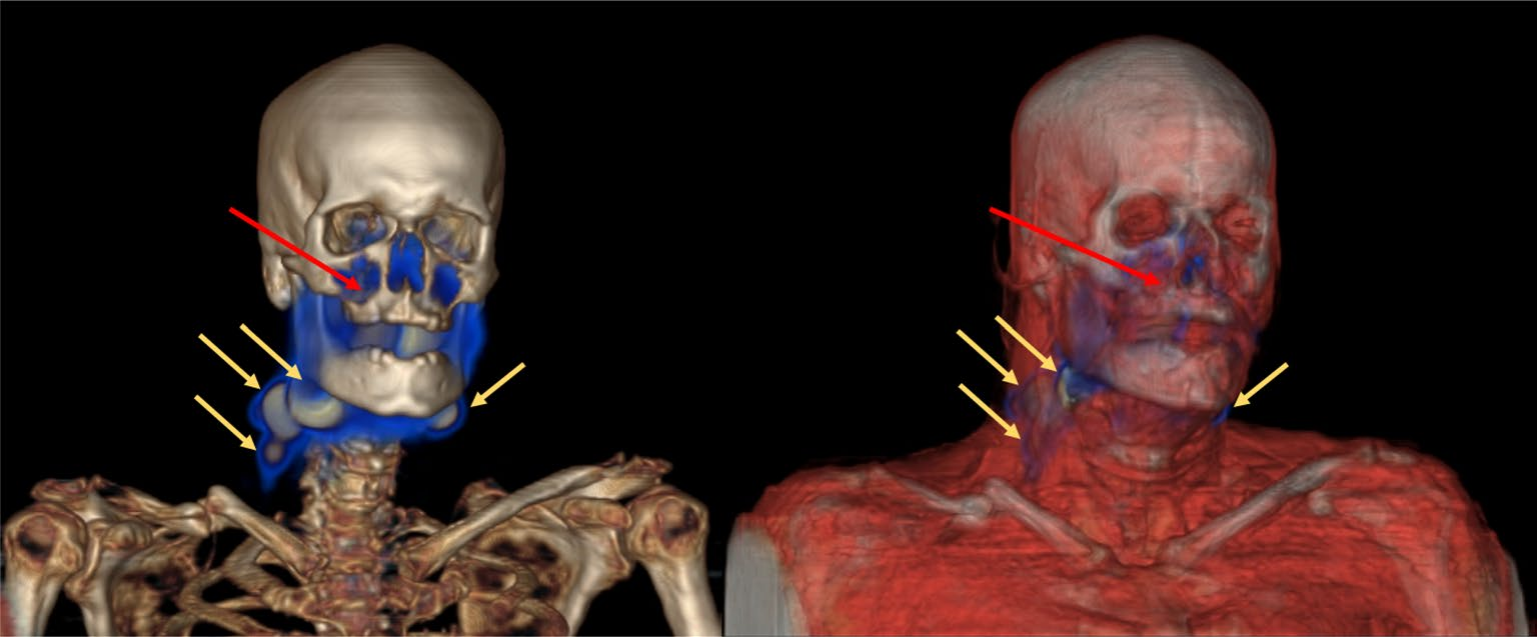
SPECT–CT shows bilateral SNs in a patient with adenocarcinoma in the soft palate, right side. Red arrow = intratumoral injection site; yellow arrows = SNs in the neck.

Radiotracer uptake consistent with SN location was detected on SPECT–CT in 33 of 39 (85%; 95% CI 70%–94%) patients. Among the six of 39 (15%) patients in whom no SNs could be detected, there were no consistent similarities in age, tumor size or time from injection of tracer to SPECT–CT. None of the 39 included patients had a previous history of surgery or radiation therapy to the head and neck area. One patient with a submandibular gland tumor received Tilmanocept instead of Nanocolloid, and SNs were successfully detected; however, as this was a single case, no comparison between the tracers was made. The median number of SNs identified per patient was 1 (0–5) for submandibular gland tumors and 1.5 (0–6) for minor salivary gland tumors. About 19 of 39 patients (49%; 95% CI 33%–65%) had SNs in one neck level only. All SNs identified on SPECT–CT were also confirmed intraoperatively using a gamma probe. SN distribution (levels I–III vs. IV–V) was analyzed in relation to tumor size (< 2 cm vs. ≥ 2 cm) and tumor site (minor salivary gland vs. submandibular gland). Fisher's exact test was used due to small cell counts, and no significant associations were observed (tumor size: *p* = 0.71; tumor site: *p* = 0.25). In patients with submandibular gland tumors (*n* = 27), no bilateral or contralateral SNs were detected. Among patients with tumors in minor salivary glands (*n* = 12), bilateral SNs were identified in two patients (tumors located in soft palate and floor of the mouth, respectively). An example of lymphatic drainage from a palatal tumor to bilateral SNs on SPECT–CT is shown in Figure 2. No adverse events related to the SN technique were seen.

### 3.4 SLNB Analysis

SLNB was performed in 16 patients. Intraoperative ICG detection with optical fluorescence imaging corresponded to gamma probe measurements. Three patients had submandibular gland tumors with nodal spread in neck levels II or III. In the other two patients, with minor salivary gland tumors in the retromolar trigone and floor of the mouth, respectively, occult metastasis was detected in neck levels II or III. Tumor location, histopathological diagnosis, T stage and neck level of positive SNs are presented in Table 3.

**TABLE 3:** Tumor location, histopathological diagnosis, T stage, and neck level of SN with micrometastasis.

Patient	Tumor location	Histopathological diagnosis	pT stage	Neck level of positive SN
1	Submandibular gland	Adenoid cystic carcinoma	T4	III
2	Submandibular gland	Adenoid cystic carcinoma	T2	II
3	Submandibular gland	Salivary duct carcinoma	T2	II
4	Minor salivary gland (retromolar trigone)	Mucoepidermoid carcinoma	T2	II
5	Minor salivary gland (floor of the mouth)	Carcinoma ex pleomorphic adenoma	T1	II (bilateral)

Abbreviations: SN, sentinel node; T stage, tumor stage.

## 4 Discussion

This study investigates the lymphatic drainage patterns and SN distribution in patients with non‐parotid salivary gland tumors, focusing specifically on submandibular and minor salivary gland tumors. Ipsilateral SN distribution was seen in all patients with tumors in the submandibular gland, whereas bilateral SN distribution was seen in patients with minor salivary gland tumors in the oral mucosa (retromolar trigone and floor of the mouth). Occult micrometastasis (positive SN) was detected in lymph nodes both in patients with submandibular and minor salivary gland tumors. Among the six patients with non‐visualized SNs, no clearly identifiable similarities in patient‐ or technique‐related factors were observed, including age, tumor size, tumor localization, prior surgery or radiation therapy, or time from tracer injection to SPECT–CT imaging. Although ultrasound‐guided tracer injection was performed, one possible explanation for the relatively high proportion of non‐visualized SNs may be suboptimal tracer injection technique or the limited sample size of the study. The existing data on the SN distribution in patients with tumors originating from the submandibular gland and minor salivary glands are still sparse. This study brings further knowledge, and the data indicates that the SN technique is feasible in this patient group.

### 4.1 Submandibular Gland Tumors

In patients with tumors in the submandibular gland, SNs were detected only ipsilaterally, involving neck levels I to IV, with no involvement of level V. Our finding is well in line with reported lymph node metastasis in cN+ patients with submandibular gland cancer, where metastases in level V are rare [19]. Consistently, a meta‐analysis by Yan et al. showed that level V also contained the lowest number of occult nodal metastases in cN0 patients undergoing elective neck dissection [20]. In our cohort, occult metastases in SNs were found in three out of 14 patients with submandibular tumors who underwent SLNB, located in levels II and III. The incidence of lymph node metastasis in submandibular gland cancer ranges in the literature from 8% to 48% [19, 21] and the rate of occult lymph node metastasis has been reported as 19.5% (mainly adenoid cystic carcinoma and mucoepidermoid carcinoma) in a meta‐analysis with 306 patients [11]. Since lymph node metastasis is an independent negative prognostic factor for survival [11, 22, 23], a known presence of microscopically disseminated disease at diagnosis is of value and may influence treatment choice and thus survival.

### 4.2 Minor Salivary Gland Tumors

In the present study, lymphatic drainage and SN distribution from minor salivary gland tumors were primarily located in levels I–III, which is well in line with previous findings [16, 18]. However, in contrast to earlier reports, we also identified one SN in level V. Bilateral SN distribution was observed in two of 12 patients. Occult metastases were detected in two cases where SLNB was performed; one of these patients had bilateral micrometastases, a finding also reported by Shilling et al. [16]. The risk of contralateral or bilateral lymph node metastasis from tumors located near the midline has also been highlighted in other studies [24]. Yan et al. reported that occult lymph node metastasis occurred in 18%–37% of minor salivary gland tumors across reviewed studies [7, 20, 25, 26, 27], and Suárez et al. found similar rates of 22%–31% in adenoid cystic carcinoma of the oral cavity and oropharynx [28]. Together with the occult SN metastases identified in our study, these data show that occult nodal disease is not uncommon in minor salivary gland tumors and underscore a potential clinical relevance of SLNB for this patient population.

### 4.3 Management of cN0 Neck in Salivary Gland Cancer

Recommendations regarding elective treatment of the neck in cN0 salivary gland cancer vary in the literature. Some authors advocate for a more aggressive approach, at least when patients harbor a high‐grade tumor [17, 18], while some are more prone to a watch‐and‐wait strategy [19, 21]. These studies are often based on salivary gland tumors as a group or specifically parotid tumors. Few studies specifically address submandibular or minor salivary gland tumors. The reported risk of occult cervical lymph node metastasis from a malignant tumor located in the parotid gland, submandibular gland, sublingual gland or in minor salivary glands varies widely in the literature [20, 25, 26, 27, 28, 29, 30, 31, 32]. In a meta‐analysis conducted by Ho et al. and Warshavsky et al., the rate of occult lymph node metastasis for salivary gland tumors in different anatomical sites varied mainly depending on histopathological tumor type and the neck level involved [11, 33, 34]. One study reported a higher risk of lymph node metastasis in adenoid cystic carcinoma if the tumor was located in a minor salivary gland compared to a major salivary gland [35].

High‐quality prospective studies comparing elective neck dissection to a watch‐and‐wait protocol are lacking, and given the low incidence of these tumors there is reason to rely on data from well‐conducted retrospective studies [4]. One should also bear in mind that the same authors found a relatively high rate of false positive lymph nodes (31/81, 38%) in their study [36], which further strengthens the indications for a selective neck level treatment with SN approach in salivary gland carcinomas.

Due to difficulties in determining the histopathological subtype accurately prior to surgery, decision‐making regarding neck management remains challenging. The incorporation of the SN technique as a diagnostic tool during primary tumor resection in cN0 cases can provide critical information on the presence or absence of neck metastasis. When combined with histopathological subtype and grading, this approach can facilitate more informed decisions regarding treatment of the neck.

Limitations of this study include the rather small cohort and the limited number of excised SNs, which reduce the generalizability. The incidence of the disease is low, which made it difficult to obtain a large cohort, especially in this single‐center setting. Tumor heterogeneity, inclusion of both benign and malignant tumors and the lack of long‐term follow‐up also limit the generalizability of the findings. However, the primary purpose of this study was to assess the feasibility and safety of applying the standard SN protocol for oral squamous cell carcinoma to tumors originating from the submandibular and minor salivary glands, and to describe their lymphatic drainage pattern. Together with our recently published work on parotid tumors [17], the data provide valuable knowledge on neck management in these patients and support the feasibility of using SPECT–CT and SN technique for salivary gland malignancies. Larger studies are needed to validate the diagnostic value of the technique for these patients.

## 5 Conclusions

SN mapping was feasible in most patients with non‐parotid salivary gland tumors, and the findings may suggest a potential diagnostic benefit in nodal staging in this patient group. However, given the small cohort size, these findings should be interpreted with caution. Further studies are needed to evaluate the diagnostic accuracy of SN mapping and its possible impact on nodal staging and recommended treatment.

## Author Contributions

**M.J.:** data curation (equal), visualization (equal), validation (equal), methodology (equal), formal analysis (equal), funding acquisition (equal), and writing – original draft (lead), writing – reviewing and editing (lead). **R.B.:** formal analysis (equal), investigation (equal), visualization (equal), methodology (equal), funding acquisition (equal), project administration (supporting), validation (equal), writing – original draft (supporting), supervision (supporting), and reviewing and editing (equal). **A.E.:** investigation (equal), visualization (equal), methodology (equal), formal analysis (equal), supervision (supporting), reviewing and editing (equal). **L.H‐.N.:** conceptualization (lead), investigation (equal), visualization (equal), supervision (equal), writing – original draft (equal), and reviewing and editing (equal). **C.G.:** conceptualization (lead), investigation (equal), data curation (equal), formal analysis (equal), visualization (equal), funding acquisition (equal), methodology (equal), validation (equal), resources (equal), software (supporting), supervision (lead), project administration (lead), writing – original draft (equal), and reviewing and editing (equal).

## Funding

The work was funded by ACTA Oto‐Laryngologica Foundation and Department of Ear Nose and Throat.

## Ethics Statement

All procedures conducted in this study adhered to the ethical standards of the institutional and national research committees, as well as the 1964 Helsinki Declaration and its subsequent amendments or equivalent ethical guidelines. Written informed consent was obtained from all participants. The study received approval from the Head of the Department of Head and Neck Cancer at Karolinska University Hospital in Stockholm, Sweden, following authorization by the National Ethical Committee (Ethical review numbers: 2019‐05211, 2022‐05287‐02, 2024‐06940‐02).

## Consent

All participants in this study were adults who provided written informed consent prior to inclusion.

## Conflicts of Interest

L.H‐.N. is a scientific review committee member of MSD. The other authors declare no conflicts of interest.

## Data Availability

The data that support the findings of this study are available on request from the corresponding author. The data are not publicly available due to privacy or ethical restrictions.

## References

[1] A. Skálová , M. D. Hyrcza , and I. Leivo , “Update From the 5th Edition of the World Health Organization Classification of Head and Neck Tumors: Salivary Glands,” Head and Neck Pathology 16 (2022): 40–53. 35312980 10.1007/s12105-022-01420-1 PMC9018948

[2] P. J. Bradley and M. McGurk , “Incidence of Salivary Gland Neoplasms in a Defined UK Population,” British Journal of Oral & Maxillofacial Surgery 51, no. 5 (2013): 399–403. 23103239 10.1016/j.bjoms.2012.10.002

[3] K. Bjørndal , A. Krogdahl , M. H. Therkildsen , et al., “Salivary Gland Carcinoma in Denmark 1990–2005: A National Study of Incidence, Site and Histology. Results of the Danish Head and Neck Cancer Group (DAHANCA),” Oral Oncology 47, no. 7 (2011): 677–682. 21612974 10.1016/j.oraloncology.2011.04.020

[4] M. Westergaard‐Nielsen , C. Godballe , J. G. Eriksen , et al., “Salivary Gland Carcinoma in Denmark: A National Update and Follow‐Up on Incidence, Histology, and Outcome,” European Archives of Oto‐Rhino‐Laryngology 278, no. 4 (2021): 1179–1188. 32691231 10.1007/s00405-020-06205-2

[5] P. Wahlberg , H. Anderson , A. Biörklund , T. Möller , and R. Perfekt , “Carcinoma of the Parotid and Submandibular Glands—A Study of Survival in 2465 Patients,” Oral Oncology 38, no. 7 (2002): 706–713. 12167424 10.1016/s1368-8375(02)00007-6

[6] M. Westergaard‐Nielsen , C. Godballe , J. G. Eriksen , et al., “Epidemiology, Outcomes, and Prognostic Factors in Submandibular Gland Carcinomas: A National DAHANCA Study,” European Archives of Oto‐Rhino‐Laryngology 280, no. 7 (2023): 3405–3413. 37052687 10.1007/s00405-023-07940-y PMC10219881

[7] C. P. Nobis , N. H. Rohleder , K. D. Wolff , S. Wagenpfeil , E. Q. Scherer , and M. R. Kesting , “Head and Neck Salivary Gland Carcinomas—Elective Neck Dissection, Yes or No?,” Journal of Oral and Maxillofacial Surgery 72, no. 1 (2014): 205–210. 23891016 10.1016/j.joms.2013.05.024

[8] E. Stennert , D. Kisner , M. Jungehuelsing , et al., “High Incidence of Lymph Node Metastasis in Major Salivary Gland Cancer,” Archives of Otolaryngology—Head & Neck Surgery 129, no. 7 (2003): 720–723. 12874071 10.1001/archotol.129.7.720

[9] T. Ettl , M. Gosau , G. Brockhoff , et al., “Predictors of Cervical Lymph Node Metastasis in Salivary Gland Cancer,” Head & Neck 36, no. 4 (2014): 517–523. 23780687 10.1002/hed.23332

[10] S.‐H. Yoo , J.‐L. Roh , S.‐O. Kim , et al., “Patterns and Treatment of Neck Metastases in Patients With Salivary Gland Cancers,” Journal of Surgical Oncology 111, no. 8 (2015): 1000–1006. 25976866 10.1002/jso.23914

[11] A. Warshavsky , I. Oz , N. Muhanna , et al., “The Rate of Occult Nodal Metastasis in Submandibular Gland Malignancies: A Case Series and Meta‐Analysis,” Oral Surgery, Oral Medicine, Oral Pathology and Oral Radiology 134, no. 3 (2022): 310–316. 35428600 10.1016/j.oooo.2022.02.004

[12] A. J. Hay , J. Migliacci , D. Karassawa Zanoni , M. McGill , S. Patel , and I. Ganly , “Minor Salivary Gland Tumors of the Head and Neck‐Memorial Sloan Kettering Experience: Incidence and Outcomes by Site and Histological Type,” Cancer 125, no. 19 (2019): 3354–3366. 31174233 10.1002/cncr.32208 PMC6744325

[13] S. Q. Qiu , G. J. Zhang , L. Jansen , et al., “Evolution in Sentinel Lymph Node Biopsy in Breast Cancer,” Critical Reviews in Oncology/Hematology 123 (2018): 83–94. 29482783 10.1016/j.critrevonc.2017.09.010

[14] V. F. Wu and K. M. Malloy , “Sentinel Node Biopsy for Head and Neck Cutaneous Melanoma,” Otolaryngologic Clinics of North America 54, no. 2 (2021): 281–294. 33743887 10.1016/j.otc.2020.11.004

[15] C. Schilling , S. J. Stoeckli , S. K. Haerle , et al., “Sentinel European Node Trial (SENT): 3‐Year Results of Sentinel Node Biopsy in Oral Cancer,” European Journal of Cancer 51 (2015): 2777–2784. 26597442 10.1016/j.ejca.2015.08.023

[16] C. Schilling , G. Gnanasegaran , S. Thavaraj , B. Vojnovic , R. Ngu , and M. McGurk , “Development of Sentinel Lymph Node Biopsy Technique in Patients With Salivary Gland Cancer Using the IDEAL Framework,” European Journal of Surgical Oncology 46, no. 11 (2020): 2029–2034. 32576478 10.1016/j.ejso.2020.05.015

[17] L. Hammarstedt‐Nordenvall , R. Bark , A. Elliot , M. Von Beckerath , and C. Gahm , “Distribution of Sentinel Nodes From Parotid Tumors‐A Feasibility Study,” Cancer Medicine 12, no. 19 (2023): 19667–19672. 37776164 10.1002/cam4.6612 PMC10587971

[18] P. Li , X. Zhang , Q. Fang , and W. Du , “Sentinel Lymph Node Biopsy in cT1‐2N0 Minor Salivary Gland Cancer in Oral Cavity,” BMC Cancer 24, no. 1 (2024): 1349. 39497070 10.1186/s12885-024-13107-7 PMC11533395

[19] M. W. Han , K.‐J. Cho , J.‐L. Roh , S.‐H. Choi , S. Y. Nam , and S. Y. Kim , “Patterns of Lymph Node Metastasis and Their Influence on Outcomes in Patients With Submandibular Gland Carcinoma,” Journal of Surgical Oncology 106 (2012): 475–480. 22457044 10.1002/jso.23100

[20] F. Yan , W. P. Lao , S. A. Nguyen , A. K. Sharma , and T. A. Day , “Elective Neck Dissection in Salivary Gland Malignancies: Systematic Review and Meta‐Analysis,” Head & Neck 44 (2022): 505–517. 34862810 10.1002/hed.26923

[21] J. G. Armstrong , L. B. Harrison , H. T. Thaler , et al., “The Indications for Elective Treatment of the Neck in Cancer of the Major Salivary Glands,” Cancer 69 (1992): 615–619. 1730113 10.1002/1097-0142(19920201)69:3<615::aid-cncr2820690303>3.0.co;2-9

[22] Y. Liu , L. Qin , R. Zhuang , X. Huang , M. Su , and Z. Han , “Nodal Stage: Is It a Prognostic Factor for Submandibular Gland Cancer?,” Journal of Oral and Maxillofacial Surgery 76 (2018): 1794–1799. 29227792 10.1016/j.joms.2017.11.012

[23] W. K. Cho , J.‐L. Roh , K.‐J. Cho , S.‐H. Choi , S. Y. Nam , and S. Y. Kim , “Lymph Node Ratio Predictive of Recurrence, Distant Metastasis, and Survival in Submandibular Gland Carcinoma Patients,” Journal of Cancer Research and Clinical Oncology 145 (2019): 1055–1062. 30806787 10.1007/s00432-019-02876-5 PMC11810156

[24] B. Baujat , S. Vergez , F. Jegoux , et al., “Lymph Node Surgery for Salivary Gland Cancer: REFCOR Recommendations by the Formal Consensus Method,” European Annals of Otorhinolaryngology, Head and Neck Diseases 141, no. 4 (2024): 215–220. 38036313 10.1016/j.anorl.2023.11.001

[25] S. Y. Lee , H. A. Shin , K. J. Rho , H. J. Chung , S. H. Kim , and E. C. Choi , “Characteristics, Management of the Neck, and Oncological Outcomes of Malignant Minor Salivary Gland Tumours in the Oral and Sinonasal Regions,” British Journal of Oral and Maxillofacial Surgery 51 (2013): e142–e147. 22939317 10.1016/j.bjoms.2012.05.004

[26] R. Xiao , R. K. V. Sethi , A. L. Feng , J. B. Fontanarosa , and D. G. Deschler , “The Role of Elective Neck Dissection in Patients With Adenoid Cystic Carcinoma of the Head and Neck,” Laryngoscope 129 (2019): 2094–2104. 30667061 10.1002/lary.27814

[27] M. Amit , S. Na'ara , K. Sharma , et al., “Elective Neck Dissection in Patients With Head and Neck Adenoid Cystic Carcinoma: An International Collaborative Study,” Annals of Surgical Oncology 22 (2015): 1353–1359. 25249259 10.1245/s10434-014-4106-7

[28] C. Suárez , L. Barnes , C. E. Silver , et al., “Cervical Lymph Node Metastasis in Adenoid Cystic Carcinoma of Oral Cavity and Oropharynx: A Collective International Review,” Auris, Nasus, Larynx 43, no. 5 (2016): 477–484. 27017314 10.1016/j.anl.2016.02.013 PMC5193158

[29] R. A. Frankenthaler , R. M. Byers , M. A. Luna , D. L. Callender , P. Wolf , and H. Goepfert , “Predicting Occult Lymph Node Metastasis in Parotid Cancer,” Archives of Otolaryngology – Head & Neck Surgery 119 (1993): 517–520. 8484940 10.1001/archotol.1993.01880170041008

[30] I. Régis De Brito Santos , L. P. Kowalski , V. Cavalcante De Araujo , A. Flávia Logullo , and J. Magrin , “Multivariate Analysis of Risk Factors for Neck Metastases in Surgically Treated Parotid Carcinomas,” Archives of Otolaryngology – Head & Neck Surgery 127 (2001): 56–60. 11177015 10.1001/archotol.127.1.56

[31] R. Kawata , L. Koutetsu , K. Yoshimura , S. Nishikawa , and H. Takenaka , “Indication for Elective Neck Dissection for N0 Carcinoma of the Parotid Gland: A Single Institution's 20‐Year Experience,” Acta Oto‐Laryngologica 130 (2010): 286–292. 19544204 10.3109/00016480903062160

[32] V. H. Lau , R. Aouad , D. G. Farwell , P. J. Donald , and A. M. Chen , “Patterns of Nodal Involvement for Clinically N0 Salivary Gland Carcinoma: Refining the Role of Elective Neck Irradiation,” Head & Neck 36 (2014): 1435–1439. 24038533 10.1002/hed.23467

[33] J. P. K. Ho , M. Mair , A. Noor , et al., “Systematic Review and Meta‐Analysis on the Incidence of Level‐Specific Cervical Nodal Metastasis in Primary Parotid Malignancies,” Otolaryngology and Head and Neck Surgery 168, no. 6 (2023): 1279–1288. 10.1002/ohn.207 36939620

[34] A. Warshavsky , R. Rosen , N. Muhanna , et al., “Rate of Occult Neck Nodal Metastasis in Parotid Cancer: A Meta‐Analysis,” Annals of Surgical Oncology 28 (2021): 3664–3671. 33175260 10.1245/s10434-020-09331-7

[35] S. Atallah , A. Moya‐Plana , O. Malard , et al., “Should a Neck Dissection Be Performed on Patients With cN0 Adenoid Cystic Carcinoma? A REFCOR Propensity Score Matching Study,” European Journal of Cancer 130 (2020): 250–258. 32008920 10.1016/j.ejca.2019.12.026

[36] M. Westergaard‐Nielsen , C. Godballe , J. Grau Eriksen , et al., “Surgical Treatment of the Neck in Patients With Salivary Gland Carcinoma,” Head & Neck 43, no. 6 (2021): 1898–1911. 33733522 10.1002/hed.26667

